# Asynchrony of directional auditory warnings and visual information in hands-on SAE level 2 automated vehicles: Analysis of driver responses

**DOI:** 10.1371/journal.pone.0336941

**Published:** 2025-11-14

**Authors:** Ali Arabian, Courtney M. Goodridge, Natasha Merat

**Affiliations:** Institute for Transport Studies, University of Leeds, United Kingdom; Beijing Technology and Business University, CHINA

## Abstract

As automated driving technology advances, the development of an efficient human-machine interface (HMI) for delivering hazard warnings becomes increasingly critical for maintaining driving safety. One example is the use of directional auditory warnings in vehicles, which are used to direct drivers’ attention, either towards a free lane to move into, or towards the location of hazards. These warnings have the potential to reduce the time it takes for drivers to take over from automation, improving overall takeover quality. Findings from lab-based studies have shown that the timing of directional auditory warnings, particularly in relation to accompanying visual cues, plays a crucial role in how effectively they are processed. Specifically, asynchrony between auditory stimuli and visual information, known as stimulus onset asynchrony (SOA), may enhance the effectiveness of auditory warnings by reducing reaction times. However, the impact of such temporal separation in real-world settings, especially during the transitions of vehicle control, is still unknown. Drivers (N = 48) were recruited for an experiment investigating how directional auditory warnings and asynchronous presentation of auditory warnings and visual information affect takeover performance from a hands-on SAE L2 automated vehicle. Results showed that directional auditory warnings reduced first steering reaction times, enhanced the accuracy of the first steering response, and resulted in smaller maximum lateral acceleration, relative to non-directional warnings. Notably, warnings that indicated the location of a free lane, and therefore a safe direction for drivers to steer towards for avoiding an obstacle, were associated with improved takeover performance and lower subjective workload. Additionally, longer SOAs were associated with reduced dominance of visual information, resulting in faster first steering response times and slight improvements in takeover quality. These findings provide evidence that directional warnings can facilitate faster and higher-quality takeover performance. Furthermore, attenuation of the visual dominance effect provides insight into how the temporal separation of auditory and visual components of audiovisual directional warnings may facilitate improved takeover performance.

## 1. Introduction

As the level of automation in road vehicles increases, operational control is the first element of driving to be disengaged [1]. In SAE Level 2 (L2) automation [2], whilst the driver is removed from the operational control loop, they must still actively monitor the road environment and intervene as needed, to maintain safety. However, there is clear evidence that driver eye movements become disrupted during hands-off SAE L2 automation, via increased gaze dispersion [3], reduced visual attention towards the road centre [4], and fewer glances to safety critical areas [5]. This reduction in operational vehicle control and/or ineffective monitoring of the road environment is thought to take drivers out-of-the-loop (OOTL) [6], which is generally associated with reductions in Situation Awareness (SA) [6,7] and can lead to poor performance following a takeover request from automation [8,9]. In order to facilitate transitions of control from automation, many vehicles are equipped with sensors that can detect elements of the road environment and provide warnings to the driver (e.g., blind-spot [10], lane departure [11], and forward collision warning systems [12], amongst many others). However, for many situations, it might not be sufficient to merely make drivers *aware* of hazards; rather, a warning may actually *guide* drivers’ attention to a specific location in order to facilitate a more effective and safe transition of operational control.

### 1.1. Directional auditory warnings in driving

As driving is a primarily visual task, several studies have suggested the use of auditory (rather than visual) warnings in driving [13,14]. The potential benefits of auditory warnings over visual warnings include the fact that individuals’ responses to auditory signals are faster than visual signals [15–17], and these warnings are ‘gaze-free’ (i.e., they do not overload the driver’s visual system) [18–20]. Tactile warnings are not as effective as auditory warnings, as their perception can be dampened by vibrations from the vehicle itself [21] and the thickness of clothing [21]. Overall, if used prudently and sparsely, auditory warnings in driving are more user-friendly and broadly preferred [22], so they are increasingly recommended and applied as directional warnings in driving [18,23–37]. In driving, directional auditory warnings that refer towards a specific location are divided into two categories. They can be used to direct drivers’ attention to the position of a free lane. This type of warning aims to facilitate a steering manoeuvre away from a potential hazard, and is often referred to as a “towards-free-lane” warnings (also known as “ipsilateral” warnings) [27,33–35,38]. Alternatively, they can inform drivers about the location of the hazard, enabling them to steer in the opposite direction to avoid a collision, termed “towards-hazard” warnings (also known as “contralateral” warnings) [27,33–35,38].

The use of directional auditory warnings has been proposed as an effective way to guide drivers’ attention to the right place at the right time during transitions of control. Recently, several studies have investigated the effects of directional auditory Takeover Requests (TORs) on drivers’ responses and performance during the transitions of control [28,32,36,37]. These studies have shown that directional auditory TORs result in faster hazard detection [32], a higher percentage of glances on the hazard [36], faster reaction times [28,32], and lower likelihood of collisions [37], when compared to non-directional auditory TORs. Despite these promising outcomes, existing research has primarily compared towards-hazard directional warnings with non-directional warnings, without examining whether guiding drivers’ attention towards a free lane or where a driver should steer to avoid an obstacle/hazard, might be equally or more effective. One study that attempted to address this gap is [26], which used a video-based driving scenario to assess the auditory towards-free-lane and towards-hazard warnings in the background of SAE L2 driving system. Results showed that participants responded faster to towards-hazard than towards-free-lane warnings. Another study by de Winter et al. [27] examined how different types of auditory warnings (i.e., towards-free-lane, towards-hazard, and non-directional) influenced driver decision-making during a simulated lane-change transition scenario. Participants were required to decide the appropriate steering response to avoid a collision with a stationary vehicle blocking the middle lane and an overtaking vehicle on the left or right side. Interestingly, while directional warnings led to faster reactions than non-directional ones, no significant differences were found between towards-free-lane and towards-hazard warnings. Additionally, eye-tracking data revealed that visual attention allocation was primarily influenced by the driving context (i.e., drivers relied on visual scanning of driving environment to respond to the lane change scenario) rather than the auditory cues. Initial and subsequent fixations tended to follow the most contextually relevant elements, such as obstacles and mirrors, suggesting that *visual information was dominant and more prominent* than the type of auditory warnings. Therefore, the hypothesis that directional auditory warnings are effective in guiding drivers’ attention to the right place was not supported by the results.

### 1.2. Cross-modal orienting of visual attention: the Colavita visual dominance effect

Attention is a selective process; this selection is pivotal because the capacity to process visual information from the environment is limited [39]. Attention can be allocated overtly (i.e., through moving one’s eyes) or covertly (i.e., by attending to a peripheral area without directing gaze [39]). Attention is guided by a range of control mechanisms (see [40,41], for a review). The two most prominent categories are top-down and bottom-up [40,42]. Whilst top-down mechanisms are shaped by the observer’s implicit goals and experience [43–45], bottom-up mechanisms are driven by the saliency of external stimuli [46,47]. The utilisation of these mechanisms to obtain visual information from the environment is crucial in many real-world tasks; perhaps none more so than driving. For example, in order to maintain adequate SA, drivers must distribute their gaze across central and peripheral road regions for moment-to-moment control [48] as well as for identifying hazards [49,50].

A key tenet of attention is that it appears to be crossmodally coordinated, with an overlap between modalities enhancing attentional processing. Overt orientating of attention is an extremely reliable behavioural reflex, even displayed in newborn babies [51,52]. Research has found that salient peripheral sounds attract attention to their location, which then influences the orientation of visual attention [53,54]. Typically, observers will respond via coordinated movements of the eyes, head, and body towards the origin of an auditory stimulus [55]. This is even the case for covert orienting, which occurs without consequential shifts in the overt receptors (i.e., the eyes). A paradigm used to investigate these cross-modal attention effects involves the presentation of a salient sound from the left or right (i.e., an auditory cue), a brief delay, followed by a visual target in either the same or opposite location. Crucially, the sounds are not necessarily predictive of the visual stimulus location. Studies have repeatedly demonstrated that these spatially non-predictive cues in one modality can attract covert attention towards its location in other modalities, not merely within the cued modality [55–59]. For example, peripheral sounds improve the discrimination of visual targets [60], and accelerate the perceived timing of visual stimuli [61].

Evidence that directional warnings are effective in guiding attention tends to come from studies that present directional sounds *before* the visual stimulus [62]. However, when auditory and visual cues are presented *simultaneously*, visual information takes precedence. One of the most famous examples of this “visual dominance” effect was found by Colavita [63]. In their [63] study, participants were asked to provide rapid discrimination responses to auditory tones and visual flashes by pressing either a “tone” key or a “light” key, respectively. While some trials featured only a tone or a light, others presented both stimuli together. Interestingly, when a tone and a light were presented together, participants responded by pressing the “light” key in almost all of the trials. Such a pre-potency for visual information has been robustly replicated [63–66]. Not only do observers respond with “light” key presses, but they are also generally slower to react to bimodal trials in comparison to unimodal trials [63,66,67]. The visual dominance effect appears to be underpinned by whether participants can establish if auditory or visual stimuli are part of the same unitary event (i.e., the unity effect). The unity effect [68,69] occurs when events perceived as coming together have a higher chance of multisensory integration, with this multisensory integration determined by the temporal separation of two events [69]. It has therefore been proposed that the Colavita effect may be attenuated when observers can distinguish between auditory and visual stimuli. Koppen and Spence [70] supported this proposal; when participants were asked to respond to an auditory target, they reacted faster when the gap between auditory and visual stimuli was largest (a Stimulus Onset Asynchrony (SOA) of −600 ms), slower for bimodal audiovisual stimuli (SOA = 0 ms), and slowest when the auditory cue was presented *after* the visual cue (SOA = +600 ms). Tilak et al. [71] and Ferris et al. [72] sought to confirm whether the performance effects of cross-modal links in spatial attention between auditory and visual modalities, observed as a function of SOA in psychophysics studies, would also replicate in the more complex environment of driving. In their manual driving simulator studies, drivers were instructed to safely navigate their vehicle and lead a convoy of vehicles through a hostile military environment. Drivers were asked to manoeuvre around roadside mines while trying to stay on the road as much as possible. When a roadside mine was detected on the left or right side, a red or green LED (mounted on either side of the monitor displaying the driving scene) illumination briefly occurred to communicate hazard’s location or free lane passage, respectively. Before the LED was illuminated, a directional auditory warning was presented either on the same side (ipsilateral) or the opposite side (contralateral) at varying SOAs. The results showed that ipsilateral auditory warnings resulted in faster reactions compared to contralateral warnings [71,72]. Additionally, as the SOA increased, the reaction time to directional auditory cues decreased. However, there were a number of limitations with these studies. Firstly, these studies have focused solely on comparing two types of *directional* auditory warnings, leaving unresolved whether these warnings are more effective than their non-directional counterparts. Secondly, although these were driving simulator studies, drivers responded to the scenarios and auditory warnings using a button press embedded in the steering wheel rather than an actual steering manoeuvre. Unlike a button press, which occurs at a single point in time, a driver’s response to a collision scenario unfolds continuously throughout the manoeuvre.

### 1.3. Rationale for the current study

Previous research has proposed that auditory cues are not only useful for guiding visual attention [73,74] but may also enhance visual processing in the cued location [61]. Much of the evidence supporting the effectiveness of directional auditory cues comes from psychophysics experiments for investigating cross-modal attention, where directional sounds are presented *before* the visual stimulus [62]. However, when auditory and visual cues are presented simultaneously, visual information takes precedence; this phenomenon was also observed in driving context [27]. Psychophysics experiments have shown that the visual dominance illustrated by the Colavita effect is attenuated with increasing SOA between the auditory and visual cues, leading to faster response to auditory cues [70]. Research has also shown this in more applied settings [71,72], however there are some limitations. Although Ferris et al. [72] and Tilak et al. [71] conducted a driving simulator study to replicate the influence of SOA on the effectiveness of directional auditory warnings, the drivers’ reaction was a button press rather than an actual steering manoeuvre. Additionally, these studies lacked comparing directional versus non-directional auditory warnings. The aforementioned limitations prompted us to conduct the current study with the aim of investigating the effects of directional auditory warnings on drivers’ responses during transitions from SAE Level 2 driving to manual control, while also highlighting the effects of audiovisual asynchrony.

## 2. Method

### 2.1. Participants

48 participants (23 female, 25 male) aged between 24 and 69 years (Mean = 41.94 ± 13.04) were recruited for the study. Two participants did not report their approximate annual mileage, or the length of driving experience. The remaining 46 participants had a valid UK driving licence (Mean = 20.22 ± 14.48, range = 2–50 years) and were regular drivers (Mean annual mileage = 7673.91 ± 3306.92, range = 5000–25000 miles). Participants were recruited via the University of Leeds Driving Simulator (UoLDS) database, supplemented by a range of social media advertisements. Criteria for participating in the study were clearly highlighted in both the recruitment emails and adverts. To be eligible, participants needed to hold a valid UK driving licence for at least two years and be regular drivers, defined as driving at least once a week and averaging around 5,000 miles per year. Participants also had to be between 21 and 70 years of age, have normal or corrected-to-normal vision and hearing, and be free from any severe mobility impairments. This recruitment approach aligns with established practices in driving simulator research (e.g., [3,75,76]), where participants are typically screened based on driving experience, licence validity and regular driving habits.

**Table 1:** The experimental design.

Warning direction	Audio file	Headphone channel	Hazard (i.e., truck) location	Driver response (No. of trials)	SOA levels (ms)	Trials per SOA
**Towards-free-lane drive (24 trials)**	“Left”	Left	Right lane	Steer left (8 trials)	0, 200, 400, 600	2 each
	“Right”	Right	Left lane	Steer right (8 trials)	0, 200, 400, 600	2 each
	“Look”	Both	Right lane	Steer left (4 trials)	0, 200, 400, 600	1 each
	“Look”	Both	Left lane	Steer right (4 trials)	0, 200, 400, 600	1 each
**Towards-hazard drive (24 trials)**	“Left”	Left	Left lane	Steer right (8 trials)	0, 200, 400, 600	2 each
	“Right”	Right	Right lane	Steer left (8 trials)	0, 200, 400, 600	2 each
	“Look”	Both	Left lane	Steer right (4 trials)	0, 200, 400, 600	1 each
	“Look”	Both	Right lane	Steer left (4 trials)	0, 200, 400, 600	1 each

**Fig 1:**
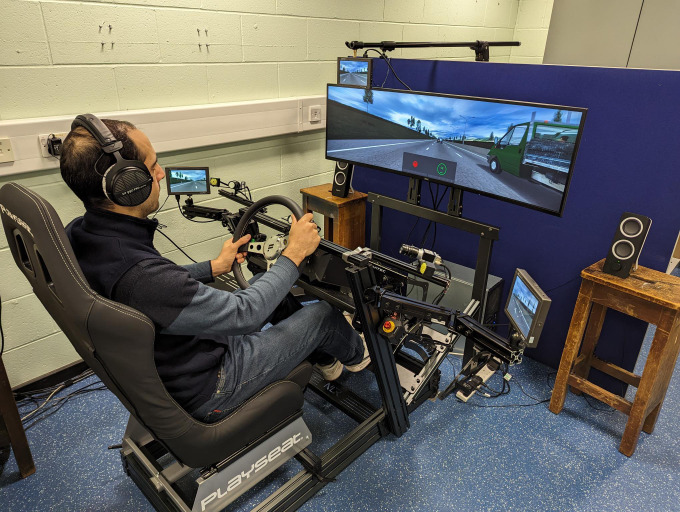
Fixed-base driving simulator set up.

### 2.2. Apparatus

The study was conducted on the University of Leeds fixed-base driving simulator, consisting of an ultra-widescreen curved 49-inch 32:9 (3840 x 1080 pixels) LED monitor, separate screens for right and left wing mirror and rear view mirror views, with FANATEC force feedback steering wheel, and pedal system (Fig 1). The accelerator and brake pedals were placed on a stable Next Level Racing® Wheel Stand DD. The simulator software was developed in-house. The simulator recorded driving data, such as acceleration and the vehicle’s lateral and longitudinal positions at a sampling rate of 60 Hz. The simulator was located in a quiet room to minimise external distractions. Participants wore a set of Beyerdynamic DT-990 Pro 250 Ohm headphones throughout the study, which was used to present the auditory warnings.

### 2.3. Experimental design

A 3 x 4 within-participants design was utilised in the current study with warning direction (towards-free-lane, towards-hazard, and non-directional), and SOA (0, 200, 400, and 600 ms) as the independent variables.

Each participant completed two drives, presented in a counterbalanced order: one with “towards-free-lane” warnings and another with “towards-hazard warnings”. The non-directional warnings were presented within each drive, as a control. The directional auditory warnings consisted of a human voice delivering the words “Left” and “Right”, presented via the headphones to participants’ left or right ear, respectively. For the non-directional cues, the word “Look” was presented simultaneously in both ears. Audibility of these words was checked with each participant at the start of the practice trials. Each drive consisted of 24 randomised trials incorporating hazard location (left vs. right), directional/non-directional warnings, and SOAs see Table 1.

### 2.4. Auditory warnings

The auditory warnings consisted of a human voice delivering a message that indicating the need to steer in a particular direction (directional) or a general message to look around (non-directional). We used the words “Left” and “Right” as the directional warnings to indicate the free lane direction in the towards-free-lane drive and the location of the hazard in the towards-hazard drive. Additionally, the word “Look” was used as the non-directional warning. Our approach aligned with the NHTSA design guidelines [77], which recommend that speech warning messages should be concise, limited to a single word or a short phrase with the fewest number of syllables. Directional (Left, Right) and non-directional (Look) audio files were created using a speech generator [ [78] spoken by English (UK) – Oliver at a speed of 1]. English (UK) was selected as the speech language because all participants were fluent English speakers, and the study was conducted in the UK. The Oliver male English voice from United Kingdom features a unique tone and accent that is characteristic of its country of origin. It demonstrates accurate pronunciation and natural intonation, making it clear and easy to understand [79]. The speed of speech is also a key factor influencing listening comprehension [80]. In several studies focused on listening comprehension, a delivery rate of 150–160 words per minute (w.p.m.) has been established as the normal or average speed for English speech [81–83]. The average speed of 150 (w.p.m) has also been used and recommended as auditory speech warnings in driving [84]. In other words, on average each word takes 400 ms to be delivered. According to this guideline, the duration of speech warnings (right, left, and look) was set to 400 ms. Previous research has commonly used warning loudness levels between 60 and 70 dB in driving scenarios. For example, Ho and Spence [18] employed auditory warnings at approximately 62–64 dB, while de Winter et al. [27] used levels around 66–68 dB. Based on this range, the warning loudness in the current study was set at 65 dB.

### 2.5. Stimulus onset asynchrony

Given the influence of SOA in attenuating the visual dominance effect, this study aimed to examine how driver responses are affected by the presence and absence of SOAs, as well as by increasing SOAs during transitions of control. Previous research has shown that the presence of an SOA between the presentation of auditory and visual cues facilitates faster reactions to directional auditory cue [70–72]. Additionally, as the SOA increases, the visual dominance effect diminishes, and the reaction time to directional auditory cues decreases. For example, a study by Koppen & Spence showed that when the SOA increases up to 600 ms, the visual dominance effect diminishes and reaction time decreases [70]. Based on this evidence, the current study included four different SOAs: 0, 200, 400, and 600 ms.

### 2.6. Driving scenario

**Fig 2:**
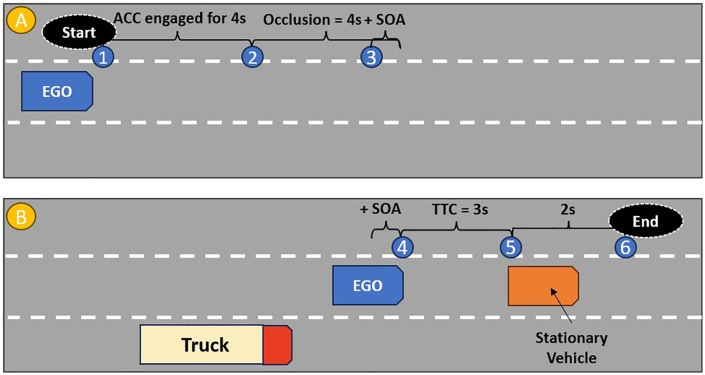
Schematic representation of the Time Windows. (A) represents the “before occlusion” and “occlusion” phase of the scenario, as follows: (1) The ego vehicle begins traveling at 60 mph in the central lane. (2) The occlusion starts, masking all visual scenes from all screens. The occlusion lasts for 4 s + the SOA, (3) The onset of the auditory warning. (B) represents the “after occlusion” phase (manual driving), as follows: (4) Following the auditory warning and the time elapsed during the SOA, the occlusion ends, (5) The moment of the crash with the stationary vehicle if the driver did not intervene, (6) If the driver overtook the stationary vehicle, they would continue travelling for a further 2 s before the end of trial.

Each experimental drive started with hands-on automated SAE Level 2 driving [2]. At the beginning of each trial, the ego vehicle was travelling at 60 mph on a three-lane highway in the centre of the middle lane. After 4 seconds of automated driving, an occlusion was introduced (for 4 s + the SOA), where all screens (main driving scene and all mirrors) were occluded with grey masking. This occlusion was implemented to manipulate the onset of the auditory warnings and visual scenes so that just before the occlusion ended (SOA = 0, 200, 400, 600 ms), one of the auditory warnings (e.g., left, right or look) was delivered through a set of headphones. The onset of the auditory warning and the onset of the SOA was simultaneous, but participants were not explicitly informed about the presence of an SOA. For example, in a trial with an SOA of 0 ms, the driving scene simultaneously appeared with the onset of the auditory warning; in a trial with an SOA of 600 ms, the driving scene appeared 600 ms *after* the onset of the auditory warning. As the occlusion ended, the automation was turned off and a stationary vehicle appeared in the middle lane ahead of the ego vehicle, blocking its path. Additionally, an offside truck, travelling at 70 mph appeared in either the left or right lane, precluding drivers from moving into that lane. This condition forced drivers to steer towards the free lane to avoid colliding with the stationary lead vehicle, within a 3-second time-to-collision (TTC) window. Regardless of driver action, the trial ended after appearance of the truck/stationary vehicle. Participants were then prompted to press a button on the steering wheel to start the next trial. Fig 2 provides a schematic representation of the time windows in the driving scenario.

### 2.7. Measurements

#### 2.7.1. Objective metrics.

Three dependent variables were analysed to determine how directional auditory warnings and the presence of SOAs affect driver response. Time to first steer (TTFS) was used to measure drivers’ reaction time. This variable was measured as the time from the end of the occlusion, until the first steering input greater than 2º was applied [85]. Maximum lateral acceleration was calculated as an indicator of control stability during the takeover process [86,87], defined as the maximum value of lateral acceleration recorded between points 4 and 5 in Fig 2. Larger maximum acceleration values indicated worse takeover quality, depicting more drastic, aggressive steering manoeuvres following a takeover [87]. Finally, to examine whether using different directional warnings and the presence of SOAs result in more accurate decisions, we tested whether participants’ first steering response (greater than 2º) was directed towards or away from the free lane. The value for this binary variable was either correct (i.e., first steer towards the free lane) or incorrect (i.e., first steer away from the free lane) [88].

#### 2.7.2. Subjective metrics.

Assessing the impact of different user interfaces, especially the directionality of TOR signals, on driver workload has been considered valuable [27,30,87,89]. Workload was assessed using the NASA-TLX scale [90], which comprises six dimensions: mental demand, physical demand, temporal demand, own performance, effort, and frustration. Following each drive, participants were requested to evaluate demand for the towards-free-lane or towards-hazard warnings, for each of these six dimensions, by placing a mark on a 20-point scale, ranging from 0–20 (very low to very high). The overall workload score was computed by averaging the responses of the six items. A nine items paper questionnaire was also used to evaluate the acceptance of the towards-free-lane and towards-hazard warnings [91]. The nine items used for this questionnaire are: (1) Useful-useless, (2) Pleasant-unpleasant, (3) Bad-good, (4) Nice-annoying, (5) Effective-superfluous, (6) Irritating-likeable, (7) Assisting-worthless, (8) Undesirable-desirable and (9) Raising alertness-sleep inducing. All items were rated using a 5-point Likert scale. Items 1, 2, 4, 5, 7, and 9 were scored in reverse for the calculation. The acceptance score was calculated by averaging responses for the nine items.

### 2.8. Procedure

Participant recruitment for this study began on 18 March 2024 and concluded on 17 April 2024. Participants were provided with written instructions and a consent form upon arrival. After reading the information sheet, they signed an informed written consent form, and their demographic information (gender, age, and driving experience) was collected. We ensured confidentiality, anonymity, voluntary participation, harmlessness, and the right to withdraw consent of all participants. All procedures were approved on 12 March 2024 by the University of Leeds Faculty Research Committee (FREC) for Business, Environment, Social Sciences (Reference code: BESS+FREC 2024-1012-1510). Participants also received a verbal explanation of the experimental procedure, the objectives of the study, and an explanation of the choice of auditory warnings used for each drive. Before start driving, participants were seated in the driving simulator and asked to adjust the seat to ensure optimal comfort and control of the vehicle. Regardless of the warning type, participants were instructed to perform a safe lane-change as quickly as possible to avoid colliding with the stationary vehicle and the offside truck. For example, in Fig 2 where the offside vehicle was travelling in the right lane, participants had to steer to the left to make an appropriate steering decision. Prior to each main drive, participants took part in a practice drive to become familiar with the equipment, driving scenario, auditory warnings, and the driving simulator dynamics. These practice drives resembled the main drives and included 12 trials. However, they did not include audiovisual asynchrony (i.e., SOA = 0). After each practice drive, participants took part in the relevant main drive. The duration of each main drive was approximately 7 minutes. After each main drive, participants took a break for about 5 minutes and were asked to fill out a post-drive questionnaire assessing their acceptance of warnings, and NASA-TLX scores. The entire experiment took approximately 60 mins, and participants were compensated £15.

### 2.9. Data analysis

Each of the two drives included 24 randomised trials regarding hazard location (left vs. right), directional and non-directional warnings, and SOAs. Data was collected from 48 participants, resulting in 2304 trials. However, only 2296 trials were analysed, with eight omitted due to technical issues. This study employed a repeated measures design, where multiple observations were collected from each participant within each condition, resulting in a hierarchical data structure. To account for the hierarchical data structure, and the multiple observations nested within each participant, we utilised a multilevel modelling approach. Given that our data were non-normally distributed, we analysed the data using Generalized Linear Mixed Models (GLMMs).

The population mean (TTFS and maximum lateral acceleration) and the log-odds (steering accuracy) were modelled via a linear combination of an intercept ($\beta_{\text{0}}$), the auditory warning type ($W,\;\beta_{W}$), the SOA ($SOA,\;\;\beta_{SOA}$), and an interaction between these variables ($WSOA,\;\beta_{W:SOA}$). The auditory warning type was parametrised as W ∈{ND,  TFL, TH} whereby non-directional warnings (ND) were the reference and were contrasted against towards-free-lane (TFL) and towards-hazard (TH) warnings. SOA was treated as a continuous variable and standardised in order to maintain model convergence.

TTFS and maximum lateral acceleration were positively skewed and thus modelled using a Gamma distribution with a log link function. The model equation for the maximum lateral acceleration and TTFS metrics were as follows:


$$Y_{ij}\;\sim \;Gamma\left(\mu_{ij},\;\sigma_{ij}\right)$$

$$log\left(\mu_{ij}\right)=\;\eta_{ij}$$

$$\eta_{ij}=\left(\beta_{\text{0}}+\beta_{{\text{0}}_{j}}\right)+\left(\beta_{W_{TFL}}W_{{TFL}_{i}}\right)+\left(\beta_{W_{TH}}W_{{TH}_{i}}\right)+\;\left(\beta_{SOA}{SOA}_{i}\right)+\left(\beta_{W_{TFL}:SOA}{W_{TFL}SOA}_{i}\right)+\;\left(\beta_{W_{TH}:SOA}{W_{TH}SOA}_{i}\right)$$

$$\beta_{{\text{0}}_{j}}\;=\;N(\text{0},\;\sigma_{\beta_{{\text{0}}_{j}}})$$

Where Y denotes the outcome metric, i specified the condition of each variable, j specified the participant.

A binomial distribution with a logit link function was used to model the steering accuracy data. The model equation was as follows:


$$Y_{ij}\;\sim \;Bernoulli\left(P_{ij}\right)$$

$$ln\;(\frac{P}{\text{1}\;-\;P})=\left(\beta_{\text{0}}+\beta_{{\text{0}}_{j}}\right)+\left(\beta_{W_{TFL}}W_{{TFL}_{i}}\right)+\left(\beta_{W_{TH}}W_{{TH}_{i}}\right)+\;\left(\beta_{SOA}{SOA}_{i}\right)+\left(\beta_{W_{TFL}:SOA}{W_{TFL}SOA}_{i}\right)+\;\left(\beta_{W_{TH}:SOA}{W_{TH}SOA}_{i}\right)$$

$$\beta_{{\text{0}}_{j}}\;=\;N(\text{0},\;\sigma_{\beta_{{\text{0}}_{j}}})$$

Where Y denotes the outcome metric, i specified the condition of each variable, j specified the participant.

All models were fitted using the lme4 R package [92]. Multicollinearity of the fixed effects was investigated by calculating variance inflation factors (VIFs) using the R package Performance [93]. All fixed effects included in the GLMMs had a VIF < 5, indicating that there was no significant collinearity among them in the models [94]. To assess which random effects should be included in the models, we started with a maximal model and compared against nested models. To maintain adequate model convergence, only random intercepts were kept for the final models. Data and analysis code can be found in the following link (https://github.com/courtneygoodridge/asynchrony_soa_takeover). For the questionnaires, differences between towards-free-lane and towards-hazard warnings were assessed using paired-samples t-tests with a significance level of 0.05.

## 3. Results

### 3.1. Time to first steer

As shown in Table 2, the GLMM showed a significant effect of warning type on drivers’ reaction time (*p* < 0.001). Post-hoc analyses revealed that towards-free-lane (M = 0.509 s, SE = 0.019) and towards-hazard (M = 0.550 s, SE = 0.021) warnings resulted in significantly faster first steering reactions (*p* < 0.001) compared to non-directional warnings (M = 0.931 s, SE = 0.035). When comparing the two directional warnings, towards-free-lane warnings led to significantly faster reactions than towards-hazard warnings (*p* < 0.001). The model also revealed that SOA had a significant negative effect on reaction time (*p* < 0.001).

**Table 2:** ANOVA table and model diagnostics for Time to First Steer GLMM.

Predictors	χ^2^	DF	*p*-value
**Intercept**	3.516	1	0.061
**Warning type**	1538.167	2	**< 0.001**
**SOA**	32.232	1	**< 0.001**
Warning type * SOA	198.754	2	**< 0.001**
**AIC**	−783.5		
**BIC**	−737.8		
**Log-likelihood**	399.8		

**Fig 3:**
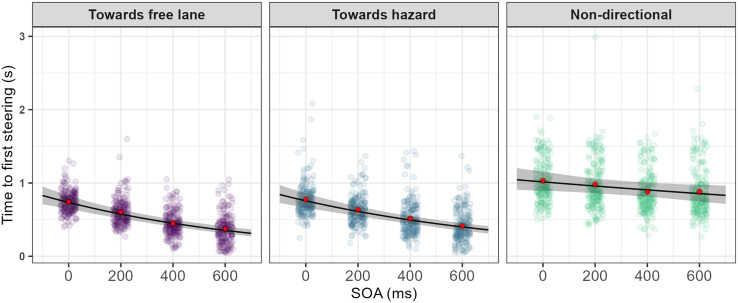
Time to first steering plotted against SOAs for the three warning directions. The black line represents the predicted mean from the model, and the grey ribbon represents 95% confidence interval bounds for the model estimate. The sample means of reaction time for each level of SOA is plotted to aid the interpretation of the model fit (red solid circles). The model estimate and 95% confidence interval bounds capture the mean response well.

Most interestingly, the TTFS model revealed a significant interaction effect between warning type and SOA (*p* < 0.001) (see Table 2), suggesting that the effect of SOA on reaction time was dependent on warning type. Post-hoc analysis of the interaction revealed that: for non-directional warnings, every 1 unit increase in standardised SOA (~224 ms on the original scale) led to a 6.44% reduction in reaction time. Conversely, for towards-free-lane and towards-hazard warnings, every 1 unit increase in standardised SOA resulted in 24.78% and 21.98% reductions in reaction time, respectively (Fig 3). In general, pairwise comparisons indicated that the SOA slopes within the towards-free-lane and towards-hazard cues had a significantly steeper gradient (more negative) than for SOA slopes for the non-directional warnings. Despite the slightly steeper SOA slope within the towards-free-lane warning contrast, the difference between the two directional warnings was not statistically significant (*p* = 0.069).

### 3.2. Maximum lateral acceleration

**Fig 4:**
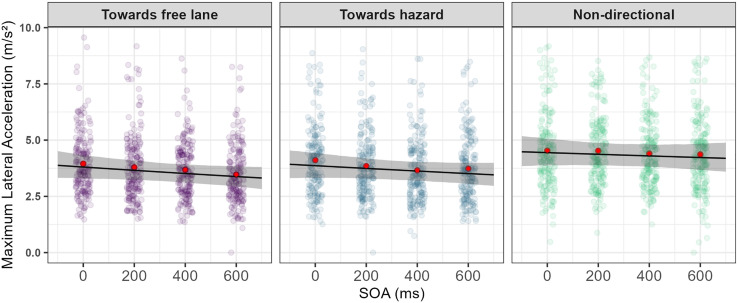
Maximum lateral acceleration plotted against SOAs for the three warning directions. The black line represents the predicted mean from the model, and the grey ribbon represents 95% confidence interval bounds for the model estimate. The sample mean of maximum lateral acceleration for each level of SOA is plotted to aid the interpretation of the model fit (red solid circles). The model estimate and 95% confidence interval bounds capture the mean response well.

As shown in Table 3, the GLMM revealed a significant effect of warning type on maximum lateral acceleration. Post-hoc analysis revealed that towards-free-lane warnings (M = 3.59 m/s^2^, SE = 0.165) and towards-hazard warnings (M = 3.68 m/s^2^, SE = 0.170) resulted in a significantly smaller maximum lateral acceleration (*p* < 0.001), compared to non-directional warnings (M = 4.33 m/s^2^, SE = 0.200). However, pairwise comparisons between towards-free-lane and towards-hazard warnings were not significant (*p* = 0.254). Furthermore, the main effect of SOA revealed that for every 1 unit increase in SOA, maximum lateral acceleration decreased by around 4%. The model revealed that the interaction between SOA and warning types was not statistically significant (Fig 4).

**Table 3:** ANOVA table and model diagnostics for Maximal Lateral Acceleration GLMM.

Predictors	χ^2^	DF	*p*-value
**Intercept**	1011.760	1	**< 0.001**
**Warning type**	144.927	2	**< 0.001**
**SOA**	2.639	1	0.104
Warning type * SOA	2.461	2	0.2921
**AIC**	7424.1		
**BIC**	7469.8		
**Log-likelihood**	−3704.0		

### 3.3. Accuracy of the first steering response

**Fig 5:**
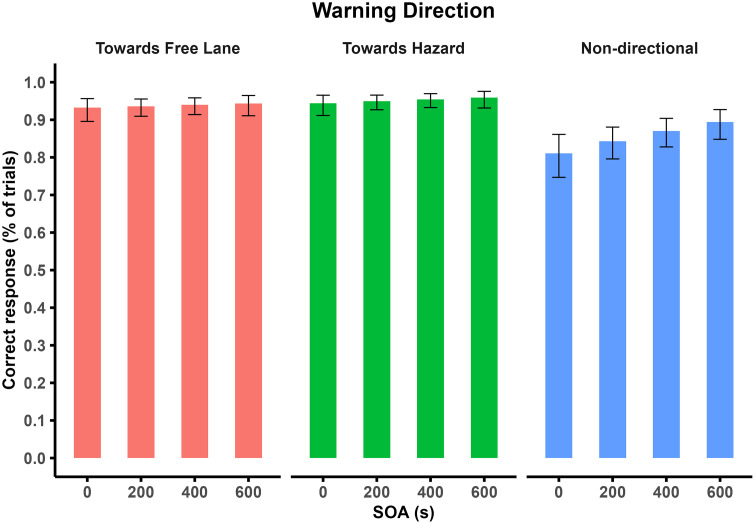
The mean with 95% CI of the accuracy of first steering response, i.e., comparing SOAs within a warning direction.

The data presented in Table 4 indicates a main effect of warning type. Towards-free-lane warnings (M = 0.938, SE = 0.011) and towards-hazard warnings (M = 0.952, SE = 0.009) led to a significantly higher likelihood of correct first steering responses (*p* < 0.001) compared to non-directional warnings (M = 0.857, SE = 0.02). The model also revealed a main effect of SOA on the accuracy of the first steering response (*p* = 0.004). The percentage change in the odds ratio showed that for every 1 unit increase in standardised SOA, non-directional, towards-free-lane and towards-hazard warnings led to a 33.90%, 8.81% and 14.78% increase in the odds of an accurate first steering response, respectively (Fig 5). However, pairwise comparisons showed no statistically significant differences between these estimates.

**Table 4:** ANOVA table and model diagnostics for response accuracy GLMM.

Predictors	χ^2^	DF	*p*-value
**Intercept**	122.117	1	**< 0.001**
**Warning type**	55.157	2	**< 0.001**
**SOA**	8.374	1	**0.004**
Warning type * SOA	1.738	2	0.419
**AIC**	1427.5		
**BIC**	1467.7		
**Log-likelihood**	−706.7		

### 3.4. Subjective metrics

The findings of the NASA-TLX questionnaire revealed that, overall, workload was significantly lower for towards-free-lane warnings than towards-hazard warnings (*p* = 0.012). Additionally, towards-free-lane warnings (M = 3.99 SD = 0.81) received slightly higher acceptance scores than towards-hazard warnings (M = 3.77, SD = 0.82, *p* = 0.064), however this did not reach stiatiscal significance, as shown in Table 5.

**Table 5:** Means, standard deviations, and results of paired-sample t-test (df = 47) of workload and acceptance ratings between warning direction types.

Questionnaires	Towards-Free-Lane Mean (SD)	Towards-Hazard Mean (SD)	t value	p value
**Overall workload**	7.44 (3.31)	8.25 (3.61)	−2.62	**0.012**
**Acceptance**	3.99 (0.81)	3.77 (0.82)	1.90	0.064

## 4. Discussion

The aim of this driving simulator experiment was to explore the effects of directional and non-directional warnings on driver takeover performance, while also considering how this was influenced by the time gap between the onset of the auditory warning and visibility of the driving scene (using a range of SOAs). Overall, results showed that drivers produced faster and higher quality takeovers when receiving directional warnings, rather than non-directional warnings, supporting results from previous SAE Level 2 studies [27]. Therefore, the directional information effectively drew drivers’ attention towards the right place at the right time when they were attempting to avoid a collision. In turn, these directional warnings also facilitated a more efficient response, with less time needed for attending to the correct location, allowing more time for an improved takeover quality.

This study also showed that the visual dominance effect attenuated as a function of SOA. When auditory signals and visual information were presented simultaneously, drivers’ first steering reactions were slower, when compared to conditions that included a temporal separation between auditory and visual information. This finding can be explained by the *unity assumption* [69,95]; which suggests that as the temporal gap between two stimuli decreases, they are perceived as one single stimulus. Additionally, the visual dominance effect suggests that individuals prioritise the visual component when processing audiovisual stimuli. Put together, it follows that when the SOA was shorter, drivers’ attention was drawn by the visual information in the driving environment (and the need to avoid a collision), potentially reducing the value of the auditory warnings [70], producing slower first steering response times. Interestingly, the attenuation of visual dominance was stronger for *directional* warnings, as indicated by the strong multiplicative effect of SOAs on steering reaction time, compared to a more linear trend for *non-directional* warnings. This may be because directional warnings facilitated the visual processing of the road environment better than non-directional cues. This is supported by a range of research indicating that auditory cues are effective in facilitating early visual processing [61,62], which in this context resulted in faster steering decisions for obstacle avoidance. These findings have important ramifications for the design of audiovisual warnings in future road vehicles, since current alerts are often presented simultaneously. A potential inference from the current study is that if a directional auditory stimulus is paired with a visual cue indicating a free lane to move into (or a hazard to avoid), it may be beneficial to delay the onset of the visual component in order to facilitate more efficient processing towards the cued location. Our findings suggest that this may result in faster and safer reactions, relative to simultaneously presented audiovisual stimuli.

Previous studies have shown that providing drivers with larger time budgets during a takeover scenario result in smoother steering manoeuvres and lower maximum lateral acceleration [96–98]. In this study, we found that directional auditory warnings resulted in smaller maximum lateral acceleration compared to non-directional warnings. This may be due to the quicker decision-making elicited by directional warnings, which took drivers’ attention to the right place at the right time, providing them with larger response-time budgets. In contrast, for non-directional warnings, responses were slower, resulting in small time budgets between the ego vehicle and stationary object, and more drastic steering manoeuvres, accompanied by higher maximum lateral accelerations, for avoiding a collision. In general, these results provide good evidence that directional cues helped drivers produce safer responses. However, the lack of significant differences between the towards free and towards-hazard lane implies that directing driver attention towards a safe location provides no additional benefits, over directing their attention towards the object they should avoid. Directional warnings also resulted in more accurate first steering responses, compared to non-directional warnings, supporting previous research [88]. However, results in this context are not consistent. For example, de Winter et al. [27] found no effect of warning direction on first steering response accuracy. This discrepancy may stem from methodological differences between the two studies, since de Winter et al. [27] utilised computer-based videos for their lane change scenarios.

Another interesting finding from our study was the effect of SOA on steering accuracy, with larger SOAs producing more accurate steering responses. Although the interaction between SOA and warning type did not reach statistical significance, this effect was slightly larger for non-directional warnings. One reason for this finding may be that directional warnings provided drivers with early information about the free lane or hazard location, which resulted in consistently high response accuracy across SOAs. Conversely, non-directional warnings lacked such information which meant that increasing the SOA provided earlier cues about the suitable response before the driving scenario appeared. Therefore, an earlier alert may have prepared drivers for their eventual takeover, resulting in more accurate steering responses [99].

Regarding subjective responses, towards-free-lane warnings were associated with lower workload and slightly higher acceptance ratings, which aligns with previous work [27]. One explanation for this finding is that a warning that guides driver action may require fewer steps before a response is given. Towards-free-lane warnings provided drivers with early information pertaining to the action they needed to complete (i.e., steer in the direction of the warning, towards the free lane). In contrast, towards-hazard warnings necessitated additional steps, such as identifying the hazard *and then* initiating a steering action. It is therefore possible that this additional step resulted in a higher subjective workload.

### 4.1. Implications

From a theoretical perspective, previous research in psychophysics has shown that providing a temporal separation between auditory and visual cues (i.e., SOA) can attenuate the visual dominance effect, leading to faster response to auditory cues [63,70]. This attenuation has also been observed in more realistic contexts, such as simulated driving studies employing a button press reaction task [71,72]. However, the current study extends these findings to a task that involves continuous visuomotor control (i.e., drivers’ steering response during transitions from automation).

From a practical perspective, the present findings highlight the importance of directionality of auditory warnings and temporal sequencing in the design of audiovisual multimodal warning systems for automated vehicles. Although multimodal (audiovisual) warnings are generally more effective than unimodal ones [100,101], our findings suggest that introducing a brief delay between directional auditory and visual components—rather than presenting them simultaneously—may facilitate more efficient attention allocation and faster driver responses. This insight offers valuable guidance for the development of next-generation HMIs that aim to enhance driver situational awareness and performance.

### 4.2. Limitations and future research

One limitation of the current study is that our results are based on speech-based auditory warnings and may not be generalisable to other types of auditory warning, since speech-based warnings typically require individuals to listen to the whole message before it is understood [102]. This additional processing can introduce delays and extend response times, particularly in time-critical situations [102]. Non-speech, abstract, warnings are also often employed in emergency scenarios, where rapid redirection of attention is essential [103,104], although the single tones used for these warnings are often difficult to localise [105]. Therefore, future studies should investigate how directional non-speech warnings, presented at different SOAs, might affect performance. This study has primarily focused on investigating the effect of directional cues on driving-related metrics, leaving it unknown whether participants followed the instructions to focus on the road. Eye tracking is the most effective way of measuring drivers’ visual attention while taking over automated driving [3,5,8,38,75,106,107]. Additional work should consider eye tracking measures to investigate drivers’ eye movement patterns in response to the directionality of warnings and the asynchrony between auditory warnings and visual information. Another element to consider is whether delivering directional warnings in a stereo nature— i.e., through two channels (“left” from both the left and right headphones) facilitates faster and better-quality takeovers compared to mono sounds, which are delivered through a single channel (“left” from only the left headphone), as used in this study. It is certainly plausible that the mono nature of the directional warnings mediated the improvement of driver takeovers, however, future research must be conducted to confirm this. Finally, this study was conducted on a static driving simulator without any motion cues. Understanding how motion affects driver response to warnings at different SOAs will be valuable, especially for implementing in real world settings.

## 5. Conclusions

This research provides valuable insights about the impact of warning directionality on driver responses, while also highlighting the effects of audiovisual asynchrony on driver performance during transitions from SAE Level 2 driving system. Both directional warnings were found to reduce first steering reaction times and improve takeover quality, compared to non-directional warnings. “Towards-free-lane” warnings were also slightly more effective, producing marginally faster reactions and lower maximum lateral acceleration values. The extent of the attenuation of the visual dominance effect was dependent on warning type. Whilst increasing SOAs resulted in a linear attenuation of visual dominance for the non-directional warnings, this was multiplicative for directional warnings. These findings highlight the potential benefits of temporally separating auditory and visual components of audiovisual directional warnings in automated vehicles, to help guide driver attention to the right location, facilitating safer takeovers.
